# Epigenetic regulation of cancer progression by EZH2: from biological insights to therapeutic potential

**DOI:** 10.1186/s40364-018-0122-2

**Published:** 2018-03-09

**Authors:** Lu Gan, Yanan Yang, Qian Li, Yi Feng, Tianshu Liu, Weijian Guo

**Affiliations:** 1 Department of Medical Oncology, Fudan University Shanghai Cancer Center, No.270, Dongan Road, Shanghai, 200032 China; 2 Department of Oncology, Shanghai Medical college, Fudan University, No.130, Dongan Road, Shanghai, 200032 China; 3 Department of Medical Oncology, Zhongshan Hospital, Fudan University, No.180, Fenglin Road, Shanghai, 200032 China

**Keywords:** EZH2, Epigenetic, Histone H3 Lys27 trimethylation, Cancer, Polycomb repressive complex

## Abstract

Enhancer of zeste homolog 2 (EZH2), a histone methyltransferase and a catalytic component of PRC2, catalyzes tri-methylation of histone H3 at Lys 27 (H3K27me3) to regulate gene expression through epigenetic machinery. EZH2 also functions both as a transcriptional suppressor and a transcriptional co-activator, depending on H3K27me3 or not and on the different cellular contexts. Unsurprisingly, numerous studies have highlighted the role of EZH2 in cancer development and progression. Through modulating critical gene expression, EZH2 promotes cell survival, proliferation, epithelial to mesenchymal, invasion, and drug resistance of cancer cells. The tumor suppressive effects of EZH2 are also identified. What is more, EZH2 has decisive roles in immune cells (for example, T cells, NK cells, dendritic cells and macrophages), which are essential components in tumor microenvironment. In this review, we aim to discuss the molecular functions of EZH2, highlight recent findings regarding the physiological functions and related regulation of EZH2 in cancer pathogenesis. Furthermore, we summarized and updated the emerging roles of EZH2 in tumor immunity, and current pre-clinical and clinical trials of EZH2 inhibitors in cancer therapy.

## Background

Epigenetic modification could regulate chromatin state and gene expression through DNA methylation and demethylation, histone modification, chromatin remodeling etc., without altering DNA sequences [1, 2]. Polycomb group proteins (PcGs), a group of important epigenetic regulators, play an important part in cell proliferation and are critical factors of pluripotency and differentiation of stem cells as well as aberrant gene expression during the malignant transformation [2, 3]. PcG proteins contain two core complexes, respectively are the maintenance complex polycomb repressive complex 1 (PRC1) and the initiation complex polycomb repressive complex 2(PRC2). PRC1 has been known to mono-ubiquitinate the histone H2A at Lys 119 through RING1A and RING1B ubiquitin ligases. PRC2 has been considered to catalyze the mono-, di-, and tri-methylation of histone H3 at Lys 27 to regulate gene transcription [3].

Enhancer of zeste homolog 2 (EZH2) is an evolutionary conserved gene identified in many species, sharing similar structural motifs and domains. As a histone methyltransferase, EZH2 has been identified as a catalytic subunit of PRC2 for tri-methylation of histone H3 at Lys 27 (H3K27me3) by SET domain in its C-terminus, which silences targeted genes and is involved in various biological functions (e.g. cell cycle, cell proliferation, cell differentiation, etc.). The role of EZH2 in cancer progression was also considered and unraveled. EZH2 is aberrantly overexpressed in various malignant tumors, such as prostate cancer, breast cancer, and ovarian cancer.EZH2 mediates H3K27me3 and functions as a critical factor in promoting tumor growth and metastasis in many malignant tumor models [3–5]. In addition, EZH2 gain or loss of function mutations have also been discovered in various cancer [4–6]. Accumulating studies indicate that inhibition of EZH2 by small molecular inhibitors or gene knockdown results in reduced cancer cell growth and tumor formation [4, 7]. Beyond playing its role in a PCR2-dependent manner as a histone modifier, EZH2 also acts in a PCR2 and histone independent manner in cancer. For example, EZH2 can methylate non-histone protein STAT3 in glioblastoma, and participate in androgen receptor-associated complexes in castration-resistant prostate cancer (CRPC) as a co-activator [5, 8, 9].The diverse functions of EZH2in cancer derive from its genetic, transcriptional, post-transcriptional and post-translational regulation in different circumstances and different types of cancer [7, 10].

In this review, we aim to discuss the molecular functions of EZH2, highlight recent findings regarding the physiological functions and related regulation of EZH2 in cancer pathogenesis. Additionally, we summarized and updated the emerging roles of EZH2 in tumor immunity, and EZH2 inhibitors in current pre-clinical and clinical trials in cancer therapy.

## Action modes of EZH2

**Fig. 1:**
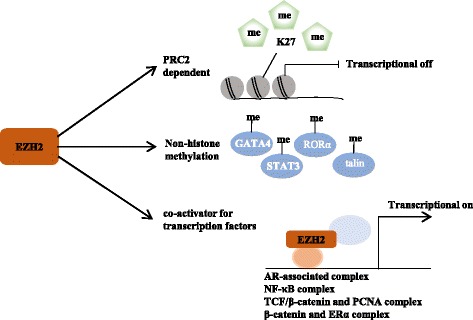
The action modes of EZH2. (1) EZH2 catalyzes H3K27me3 dependent on PCR2, which contributes to transcriptional silencing. (2) EZH2 is also capable of methylating several non-histone protein substrates (e.g. STAT3, GATA4, talin, and RORα), which contributes to both transcriptional silencing and transcriptional activation. 3) EZH2 also has a PRC2-independent role in transcriptional activation, acting as co-activator for transcription factors, such as AR-associated complex, NF-κB signaling, TCF/β-catenin and PCNA, and β-catenin and ERα

As important epigenetic regulators, there are two families of PcG complexes identified in mammals, PRC1 and PRC2. The PRC1 complex catalyzes the monoubiquitylation of histone H2A and is composed of several subunits, such as PCGF, HPH, CBX, and RING1 paralog groups, whereas the PRC2 complex contributes to the methylation of H3K27 and mainly consists of EED, SUZ12, RbAp46/48, and either EZH1 or EZH2 [2, 3]. As a histone methyltransferase, EZH2 is responsible for the H3K27 methylation. Human EZH2 gene is located on the long arm of chromosome 7 at 7q35 and encodes a 746 amino acids protein. EZH2 organizes into several domains: the conserved SET domain at the C-terminus functions for maintaining histone methyl transferase (HMT) activity, CXC domain and ncRBD domain are required for interactions with other PRC2 components and regulatory proteins [2, 3]. Accumulating studies have demonstrated that EZH2 participates in many diverse biological processes and displays different modes of actions. Here, we will firstly briefly summarize the known archetypes of molecular functions of EZH2: a dual-faced molecule acting both as a transcriptional suppressor and a transcriptional co-activator, depending on its interaction with other PRC2 components or not and in different cellular contexts (Fig. 1).

### PRC2-dependent H3K27 methylation for gene silencing

EZH2 is essential for epigenetic gene silencing. EZH2 catalyzes H3K27 trimethylation (H3K27me3) in the nucleus and then PRC1 binds to monoubiquitinated histone H2A at lysine 119 (H2AK-119ub1) and H3K27me3. This complex then mediates chromatin compaction followed by transcriptional repression of downstream genes [11].

### PRC2-dependent non-histone protein methylation for gene silencing

EZH2 also functions in the cytosol to methylate non-histone proteins in a PRC2 complex dependent way. EZH2 can directly methylate cardiac transcription factor GATA4 at Lys299, thereby attenuating p300-mediated GATA4 acetylation and promoting GATA4 transcriptional repression and gene silencing [12]. EZH2 was also shown to bind to and methylate RAR-related orphan receptor alpha (RORα) at Lys38 to promote DDB1/DCAF1/CUL4 ubiquitin ligase complex-mediated RORα ubiquitination and lead to RORα target gene silencing [13]. Gunawan et al. showed that EZH2 directly methylates talin, a key regulatory molecule in cell migration, to disrupt the binding of talin to F-actin and thereby promotes the turnover of adhesion structures [14].

### PRC2-independent gene transactivation

In addition to transcriptional repression, emerging researches have shown that EZH2 methylates non-histone targets or directly interacts with other proteins to activate downstream genes in a PRC2-independent manner. Kim et al. found that AKT phosphorylates EZH2 at Ser21. The phosphorylated EZH2 directly binds to and methylates STAT3, leading to enhanced STAT3 activity by increasing phosphorylation of STAT3 at Y705 and thereby promotes the tumorigenicity of glioblastoma and glioblastoma stem-like cells (GSCs) [8].

The phosphorylated EZH2 also acts as a co-activator of critical transcription factors. Xu and colleagues demonstrated that EZH2 acts as a coactivator of androgen receptor-associated complexes to support castration-resistant prostate cancer (CRPC) growth, suggesting novel combination therapies for the treatment of metastatic, hormone-refractory prostate cancer [9]. Lee ST and colleagues identified that EZH2 binds to the NF-κB components to form a ternary complex leading to transcriptional activation of its downstream target genes in ER-negative basal-like breast cancer cells [15]. Similarly, EZH2 is also found to act as a co-activator of other critical transcription factors, such as TCF/β-catenin and the DNA repair protein PCNA-associated factor in colorectal cancer (CRC), β-catenin and ERα in breast cancer, to promote the transcription of their target genes, respectively [16, 17].

Another study by Yan et al. shows that ectopic expression of an EZH2 mutant lacking HMTase activity in NKTL cell lines rescued the tumor growth inhibition resulting from depletion of endogenous EZH2, indicating that the growth promoting function of EZH2 is independent of its HMTase activity [18]. However, further studies are still needed to uncover the detailed mechanism.

### Dysregulation of EZH2 in cancers

**Fig. 2:**
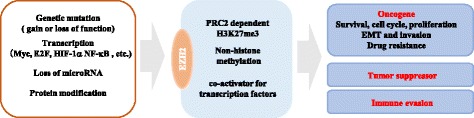
The physiological functions and related regulation of EZH2 in cancer pathogenesis. The EZH2 expression and activity in cancer cells is regulated at genetic, transcriptional, post-transcriptional and post-translational levels, which leads to diverse functions of EZH2. EZH2, mediating gene transcriptional silencing and activation, promotes cell survival, proliferation, epithelial to mesenchymal, invasionand drug resistance of cancer cells. The tumor suppressive effects of EZH2 are also identified in several cancers, especially T-ALL, pancreatic cancer and clear cell renal carcinoma. What’s more, EZH2 has decisive roles in T and NK cells mediated immune evasion

As various functions of EZH2 were discovered, it has become understandable that EZH2 plays critical roles in the development and progression of various cancers. The significance of EZH2 in cancer was firstly realized in 2002 when Varambally and colleagues elucidated the association betweenEZH2 and prostate cancer prognosis. It was found that the upregulation of EZH2is associated with advanced stage and poor prognosis in prostate cancer [19]. Subsequent studies showed that EZH2 is upregulated in various solid malignancies including lung, hepatocellular, colorectal, breast and pancreatic cancer etc., and is shown to have prognostic significance in a variety of solid cancers [3, 4]. Unsurprisingly, the biological functions of EZH2 in different kinds of tumor cells are under intense investigation. However, both overexpression and loss-of-function mutations of EZH2 gene being detected in myelodysplastic syndrome (MDS) and acute myeloid leukemia (AML) suggests that EZH2 can function as tumor suppressor gene or as an oncogene in myeloid malignancies. In addition to the expression and function of EZH2 in cancer cells, recent studies also revealed that EZH2 might have critical roles in tumor immunity. Here, we will systematically discuss the functions of dysregulated EZH2 in cancers as below (Fig. 2).

### Cell cycle, proliferation and apoptosis

EZH2 is involved in regulation of cell cycle progression and dysregulation of EZH2 accelerates cell proliferation and promotes survival, resulting in cancer development, such as CRC, melanoma and breast cancer [20–22]. For example, EZH2 inhibition by its inhibitor, DZNep or gene knockdown, can induce autophagy and apoptosis in CRC cells in vitro [20]. EZH2 inhibition in cholangiocarcinoma cells enhances cell apoptosis and arrests cells in the G1 phase, characteristized by elevated levels of p16 and p21 [23].

### Epithelial to mesenchymal transition (EMT) and invasion

Strong evidence demonstrated that EZH2 could promote EMT by interacting with SNAIL1 and down-regulating the expression of E-cadherin [24, 25]. EZH2 can also induce the gene silencing of Disabled Homolog2-Interacting Protein (DAB2IP) to regulate EMT and metastasis in CRC [26]. In addition, EZH2 is demonstrated to promote tumor cell invasion. Yi et al. found that EZH2 inhibitsTIMP2 expression via H3K27me3 and DNA methylation, by which mean it relieves MMP repression and facilitates migration and invasion of ovarian cancer cells [27]. Under hypoxia, activation ofHIF-1α results in inactivation of PRC2 and the release of EZH2. Released EZH2 participates in the EZH2/Forkhead box M1 (FoxM1) complex and induces MMP expression and invasion in triple-negative breast cancer (TNBC) [22]. As mediating the activation of EMT and invasion, EZH2 could increase metastasis in many cancers, such as CRC, melanoma, and breast cancer [22, 26, 28].

### EZH2-mediated drug resistance

Despite of the development of cancer therapy, the drug resistance of cancer remains a serious problem. The critical roles of EZH2 in drug resistance of cancer cells were also identified. Several studies showed that EZH2 overexpression enhances chemoresistance in small cell lung cancer, head and neck squamous cell carcinoma (HNSCC) and glioblastoma [29–31]. In small cell lung cancer, EZH2 mediated H3K27me3 suppressed the expression of SLFN11, a factor implicated in DNA-damage repair deficiency, leading to chemoresistance [29]. In glioblastoma, EZH2 promotes the expression of ABC transporter MDR, MRP and BCRP to strengthen chemoresistance [31]. An EZH2 inhibitor combined with standard cytotoxic therapies suppressed emergence of acquired resistance and augmented chemotherapeutic efficacy in both chemosensitive and chemoresistant models.

Acquired and intrinsic resistance to receptor tyrosine kinase inhibitors (RTKi) represents a major hurdle in improving the management of clear cell renal cell carcinoma (ccRCC). Adelaiye et al. highlighted EZH2 as a rational target for therapeutic intervention in sunitinib-resistant ccRCC. Modulating EZH2 expression or activity suppressed phosphorylation of certain RTK, restoring the anti-tumor effects of sunitnib [32]. Inhibitors against poly (ADP-ribose) polymerase (PARP) are promising targeted therapy agents currently used to treat BRCA-mutant ovarian cancer and are in clinical trials for other cancer types, including BRCA-mutant breast cancer. Yamaguchi et al. demonstrated that EZH2 regulates the sensitivity of cancer cells to PARP inhibitors (PARPi). Upon DNA damage, PARP1 interacts and PARylates EZH2, inducing PRC2 complex dissociation and EZH2 downregulation. When PARPi is used, alkylating DNA damage-induced EZH2 downregulation would be disturbed, and EZH2-mediated gene silencing and cancer stem cell property are enhanced, leading to decreased sensitivity of PARPi [33]. However, in acute myeloid leukemia (AML), loss of the histone methyltransferase EZH2 and subsequent reduction of histone H3K27 trimethylation is identified as a novel pathway of acquired resistance to tyrosine kinase inhibitors (TKIs) and cytotoxic drugs in AML. Suppression of EZH2 protein expression reduces chemoresistance of AML cell lines and primary cells in vitro and in vivo. Low EZH2 level results in derepression of HOX genes, and knockdown of HOXB7 and HOXA9 in chemoresistant cells is sufficient to improve cell sensitivity to TKIs and cytotoxic drugs [34].

### Tumor suppressive roles of EZH2

Although the oncogenic roles of EZH2 in many cancer types have been reported, the tumor suppressive roles of EZH2 were also identified. Suppression of EZH2 promotes cancer progression in some cancer types. T cell acute lymphoblastic leukemia (T-ALL) is an immature hematopoietic malignancy driven mainly by oncogenic activation of NOTCH1 signaling. Ntziachristos et al. demonstrated loss-of-function mutations and deletions of the EZH2 and SUZ12 genes in 25% of T-ALLs. Activation of NOTCH1 specifically induces loss of the repressive mark H3K27me3 by antagonizing the activity of PRC2, promoting cancer progression of T-ALL [35]. Mallen-St et al. found that loss of EZH2 resulted in impaired pancreatic regeneration and accelerated KRAS (G12D)-driven neoplasia, implicating that EZH2 restricted cancer progression via homeostatic control of pancreatic regeneration [36]. In clear cell renal carcinoma, loss of PRC2-dependent H3K27me3 in cancer cells activates HIF-driven chemokine (C-X-C motif) receptor 4 (CXCR4) expression in support of chemotactic cell invasion, leading to cancer metastasis [37].

### Emerging roles of EZH2 in tumor immunity

Aberrant EZH2 expression in cancer cells can help them to modulate immune response and immunotherapy. Peng et al. showed that ovarian tumor EZH2 and DNA methyltransferase 1 (DNMT1) are negatively associated with tumor-infiltrating CD8^+^T cells and patient outcome. EZH2-mediated H3K27me3 and DNMT1-mediated DNA methylation repress the tumor production of T helper 1 (Th1)-type chemokines CXCL9 and CXCL10, and subsequently determine effector T-cell trafficking to the tumor microenvironment. Treatment with epigenetic modulators increases tumor infiltration of effector T cell, slows down tumor progression, and improves the therapeutic efficacy of PD-L1 checkpoint blockade and adoptive T-cell transfusion in tumor-bearing mice [38]. A similar effect of EZH2 was also observed in colorectal cancer. Inhibition EZH2 in colorectal cancer cells augmented CXCL9 and CXCL10 expression to affect the infiltration of effector T cells in tumor [39]. Besides, Zingg et al. showed that during anti-CTLA-4 or IL-2 immunotherapy in mice, intratumoral tumor necrosis factor-αproduction and T cell accumulation results in increased Ezh2 expression in melanoma cells, which in turn silences their own immunogenicity and antigen presentation. Ezh2 inactivation reversed this resistance and synergized with anti-CTLA-4 and IL-2 immunotherapy to lead to intratumorally accumulation of IFN-γ-producing PD-1^low^ CD8^+^ T cells and PD-L1 downregulation to suppress melanoma growth [40].

Aberrant EZH2 expression and activity in immune cells in the tumor microenvironment affects tumor progression and therapy. Accumulating evidences have shown that EZH2 has direct roles on T cell response. EZH2 expression in naïve T cells promotes survival, proliferation and function of effector CD4^+^ and CD8^+^ T cells but inhibits Th1 and Th2 differentiation [6]. Therefore, EZH2 inhibition in T cells may suppress survival, expansion and effect of tumor-specific effectors T cell to inhibit anti-tumor immunity. Indeed, Zhao et al. demonstrated that EZH2 has a key role for the function of tumor-specific effector T cells, that cancer cells evaded tumor surveillance by targeting T-cell specific expression of EZH2 in the tumor microenvironment. The percentage of EZH2^+^ CD8^+^ T cells in ovarian cancer tissues is a stronger predictor of overall and progression-free survival as compared to the percentage of CD8^+^ T cells. Ovarian cancer cells could induce expression of specific micro-RNAs in CD4^+^ and CD8^+^ T cells of the tumor microenvironment, which suppressesEZH2 expression and decreases their survival and immune function. Inhibition of EZH2 in tumor-specific T cells increases the tumor burden and the metastatic potential in mice models of ovarian cancer [41]. Long et al. identified that miR-26a expression is elevated in CTLs responding to tumor microenvironment secretome stimulation, miR-26a inhibits EZH2 to impair CTL function, indicating miR-26a-EZH2 axis as a novel target to improve the efficacy of CTL-based cancer immunotherapy [42].

Besides, EZH2 is considered critical for the recruitment and immunosuppression function of activated regulatory T cells (Tregs) at the sites of inflammation, and EZH2-deficient Tregs fail to protect mice from the development of autoimmunity in a model of naïve T cell-mediated colitis [43, 44]. It has been documented that Tregs can be induced in tumor microenvironment and display negative impact on anti-tumor immunity. Therefore, it is of particular interest to investigate for the regulation of EZH2 expression in Tregs in tumor microenvironment.

A recent study by Yin et al. has provided insight to an epigenetic-based mechanism regulating natural killer (NK) cell development and NK-based cancer immunotherapies. Inhibition of EZH2 expression or activity in hematopoietic stem and progenitor cells (HSPCs) gives rise to increased NK precursors and mature progeny, which displays enhanced cytotoxicity against tumor cells with up-regulation of IL-15R (CD122) and the NKG2D-activating receptor [45]. Gunawan et al. demonstrated that EZH2regulated the integrin signaling and adhesion dynamics of dendritic cells (DCs) to promote the development of experimental autoimmune encephalomyelitis [14], while Donas et al. found that inhibition of H3K27 demethylation induced tolerogenic DCs to inhibit inflammation and the development of experimental autoimmune encephalomyelitis [46]. These indicate a potential role of EZH2 in DC function, and whether EZH2 regulates DC-based cancer immunotherapies is worth further investigation. Another innate immune cell macrophage in tumor microenvironment, named tumor-associated macrophages (TAMs) can promote tumor growth directly, by favoring tumor cell proliferation and survival, and indirectly, by creating an immunosuppressive microenvironment. Qiao et al. demonstrated that EZH2 mediated H3K27me3 promotes IFN-γ-induced macrophage activation and inflammatory response [47]. However, how the dysregulated EZH2 in TAM participates tumor progression is still unknown.

### Regulations of EZH2 in cancer

As shown above, EZH2 has diverse functions in different types of cancer. Accumulating evidences have shown that the expression and activity of EZH2 in cancer cells can be regulated at genetic, transcriptional, post-transcriptional and post-translational levels, which may explain these diverse functions of EZH2 (Fig. 2).

### Mutation of EZH2

Recent studies have shown that somatic mutations in *EZH2* gene are observed in specific cancer types. Heterozygous point mutations affecting tyrosine 641 (Y641) within the C-terminal catalytic SET domain of EZH2 have been identified in B-cell lymphomas [diffuse large B-cell lymphoma (DLBCL) 22%; follicular lymphoma 7–12%] [48, 49]. Functional analysis has demonstrated that this mutation mediates gain-of-function of EZH2 enzymatic activity leading to increased levels of H3K27me3 and resulting in suppression of gene expression (e.g. TCF4, FOXP1, TCL1A, BIK, RASSF6P, CDKN1A) in lymphomas [50–52]. Otherwise, Souroullas et al. demonstrated that somatic EZH2 gain-of-function mutation (Y641F) in lymphoma and melanoma induces a vast reorganization of chromatin structure, inducing both repression and activation of polycomb-regulated loci. EZH2 mutation (Y641F) globally increased the abundance of H3K27me3 in lymphoma and melanoma, but also caused a widespread redistribution of this repressive mark, including a loss of H3K27me3 that was associated with increased transcription at many loci [53].

In contrast, EZH2 inactivating deletion, frameshift, nonsense and missense mutations have been identified in myelodysplastic syndromes (MDS), myeloproliferative neoplasms (MPN), MDS-MPN overlap disorders and T-cell acute lymphocytic leukemia [54–56], implicating that EZH2 loss-of-function is associated with development of malignancy and EZH2 may function as a tumor suppressor. Consistently with these findings in patients, mice lacking EZH2 gene have enhanced initiation and progression of Runx1-mutant MDS [57]. These studies suggest that the potential tumor suppressive effects of EZH2 should be considered during therapeutic application of EZH2 inhibitors.

### Transcriptional regulation of EZH2

EZH2 has been considered to be overexpressed in many cancer types and to be transcriptionally regulated by oncogenic signaling to promote cancer cell proliferation and disease progression. EZH2 expression is correlated with Myc expression in prostate cancer. Myc binds to EZH2 promoter and directly activates its transcription [58]. E2F, another cell cycle regulator, positively controls EZH2 transcription through its direct binding on EZH2 promoter upon Rb/RB1 phosphorylation in bladder and small cell lung cancer [59]. ANCCA, a co-activator of androgen receptor (AR), can bind E2F and enhance E2F-mediated EZH2 transcription in prostate cancer cells [60, 61]. SOX4, one of the key regulators of stem cells, directly regulates the expression of EZH2 mRNA to promote EMT [28].

Besides, many other transcription factors have also been identified to affect the expression of EZH2 gene, including EWS-FLI1 in Ewing tumors [62], MEK- ERK-ELK1 in pancreatic cancer, triple negative and ERBB2 positive breast cancer [63, 64], KRAS mutations and downstream ERK or Akt in non-small cell lung cancer [65], hypoxia-induced HIF-1α in breast cancer [66], and NF-κB in T-cell leukemia [67]. All these findings provide better understanding of EZH2 regulation at the transcriptional level and allow therapeutic combinations of pathway targeting agents and EZH2 inhibitors to achieve maximum therapeutic benefit in cancers.

### Post-transcriptional regulation of EZH2

The expression of EZH2 in cancer is also regulated by post-transcriptional mechanisms through microRNAs. These microRNAs can bind to the EZH2 mRNA 3’UTR and modulate its stability, integrity and translation, thereby affecting the levels of EZH2 protein. In specific cancer types, downregulation of these microRNAs leads to EZH2 overexpression and subsequent H3K27me3 accumulation to promote tumor progression. In particular, miR-26a and miR-101 are the most demonstrated as negative regulators of EZH2. Wong et al. firstly found that miRNA-26a, an up-regulated miRNA during myogenesis, binds to the EZH2 mRNA 3’UTR and inhibits its expression in myoblasts [68]. The following studies identified that miR-26a acts as a tumor suppressor, and directly targets and regulatsEZH2 at a post-transcriptional level in cancer cells to inhibit the development of hepatocellular cancer, Burkitt lymphoma, rhabdomyosarcoma, nasopharyngeal carcinoma, and breast cancer. Similarly, Xiaoping et al. demonstrated that downregulation of miR-101 in glioblastoma cells promotes tumor angiogenesis, cell proliferation and invasion by increasing EZH2-mediated overexpression of cytoplasmic polyadenylation element-binding protein 1 (CPEB1) [69, 70].

Other microRNAs (e.g. miR-126, miR-138, miR-32, miR-506, miR-137) are also reported to directly target EZH2 in different types of cancer and displayed different functions. For example, miR-126 and miR-138 directly target EZH2 transcript and increase the sensitivity of osteosarcoma cells and gastric cancer cells to chemotherapy [71, 72]. miRNA-32, miR-137 and miR-506 also directly target EZH2 transcript and suppress tumor proliferation, angiogenesis and metastasis in melanoma, colon cancer and glioblastoma [73–75].

### Post-translational regulation of EZH2

In addition to transcriptional regulation and miRNA-mediated post-transcriptional regulation, the expression and activity of EZH2 can be regulated at the post-translational level, such as protein phosphorylation, ubiquitinylation, palmitoylation, etc.

AKT1-mediated phosphorylation of EZH2 allows it to act as a coactivator of androgen receptor-associated complexes, promoting the expression of androgen receptor-target genes in the absence of androgen and the development and progression of castration-resistant prostate cancer (CRPC) [9]. The phosphorylated EZH2 also can directly bind to and methylate STAT3, leading to enhanced STAT3 activity by increased tyrosine phosphorylation of STAT3 and thereby promote the tumorigenicity of glioblastoma stem-like cells (GSCs) [8]. Cyclin-dependent kinases 1 and 2 (CDK1/2) phosphorylate EZH2 at T492 disrupts the PRC2 assemblies and decreases EZH2 activity, while phosphorylation of EZH2 at T350 and T492 promotes EZH2 ubiquitinylation and degradation, suggesting its decreased methyltransferase activity [76, 77]. While CDK-mediated EZH2 phosphorylation is critical for cancer-cell invasion and osteogenic differentiation of mesenchymal stem cells, further investigation is needed toreveal the role of this interaction in the fate and function of cancer cells. Another study by Yang et al. showed that cyclin E/CDK2 phosphorylates EZH2 at T416 (pT416-EZH2) and enhances the ability of EZH2 to promote triple negative breast cancer cell migration/invasion, tumor sphere formation and in vivo tumor growth [78].

Wang et al. also identified NIMA-related kinase 2 (NEK2) as a functional binding protein of EZH2 that plays a critical role in the posttranslational regulation of EZH2 protein in GSCs. NEK2 is among the most differentially expressed kinase-encoding genes in GSC-containing cultures, and is required for in vitro clonogenicity, in vivo tumor propagation and radioresistance. Mechanistically, the formation of a protein complex comprising NEK2 and EZH2 in glioma spheres phosphorylates and then protectsEZH2 from ubiquitination-dependent protein degradation in a NEK2 kinase activity-dependent manner [79]. Besides, Yan et al. demonstrated that JAK3, a tyrosine kinase activated downstream of cytokine receptors, phosphorylates EZH2 on tyrosine residue 244, altering EZH2 activity and promotes the survival and proliferation of NK/T-cell lymphoma cells [80].

EZH2 can also be regulated by protein ubiquitination. Sahasrabuddhe et al. identified EZH2 as a novel inter actor and substrate of the SCF E3 ubiquitin ligase FBXW1. FBXW1ubiquitinatesEZH2, and Jak2-mediated phosphorylation on Y641 directsFBXW1-mediated EZH2 degradation, thereby controlling H3K27me3 activity and lymphoma pathogenesis [81]. Jin et al. identified another E3 ubiquitin ligase, FBW7that targets EZH2 in pancreatic cancer cells. The expression of EZH2 protein is negatively correlated with FBW7 protein levels in a cohort of human pancreatic cancer specimens, and FBW7 suppresses EZH2 activity and inhibits tumor migration and invasion via degradation of EZH2 in pancreatic cancer cells [82].

Protein S-palmitoylation is a reversible posttranslational modification in proteins with fatty acids that is regulated by protein acyltransferases (PAT), characterized by a conserved catalytic domain containing an Asp-His-His-Cys (DHHC) motif [83]. A recent study found that the gene-encoding zinc finger DHHC-type-containing (ZDHHC) 5 directly palmitoylates EZH2 to increase EZH2-mediated H3K27me3 but not EZH2 expression, thereby promoting tumorigenicity of GSCs [84]. Yamaguchi et al. reported that during DNA damage, PARP1 mediates PARylation of EZH2, inducing PRC2 complex dissociation and EZH2 downregulation. Inhibition of PARP by PARPi attenuates alkylating DNA damage-induced EZH2 downregulation, thereby promotes EZH2-mediated gene silencing and cancer stem cell property compared with PARPi-untreated cells [33].

Collectively, all these findings strongly suggest that posttranslational regulation of EZH2 might be of particular importance with regards to its diverse and complex implication in cancer development and growth.

### Targeting EZH2 for cancer therapy

EZH2 widely regulates gene expression affecting several physiological functions, especially cancer progression. Thus, EZH2 is a potential target for cancer therapy, and EZH2 inhibitors have been under intense pre-clinical and clinical investigation.

3-deazaadenosine A (DZNep) is a non-specific EZH2 inhibitor which decreases the protein level of EZH2 and the H3K27me3 marks, thereby exhibiting anti-tumor effects in various malignancies. However, DZNep was reported to have efficacy but also toxicity in animal models [85].

Recently, some studies referred that targeting histone methylation might be a promising approach for cancer therapy, for example, lysine-specific demethylase 1 inhibition increased histone H3lysine4 methylation and finally induced apoptosis and differentiation of stem-like leukemia cells [86, 87]. In 2012, potent, highly selective S-adenosyl-methionine-competitive small molecule inhibitors of EZH2 methyltransferase activity, GSK126 [88] and EPZ005687 [89], were developed.GSK126 and EPZ005687 can bind to wild-type and Y641 mutant EZH2, leading to decreased H3K27me3 level and upregulation of expression of the silenced gene transcription, thereby effectively inhibiting the proliferation of EZH2 mutant DLBCL both in vitro and in xenograft mice models [88, 89]. GSK926 [90] and GSK-343 [91] are also exhibited to inhibit EZH2 activity to suppress H3K27me3 level in breast and prostate cancer cells. An oral inhibitor of EZH2, EPZ6438 (tazemetostat), reportedly decreases the levels of H3K27me3 to reduce tumor growth in EZH2 mutant non-Hodgkin lymphoma [92]. EPZ6438is currently under evaluation in a phase 1/2 clinical trial in patients with B-cell lymphomas and advanced solid tumors (NCT01897571), provided with encouraging preliminary signs of clinical activity. Besides, this compound is being further evaluated in a phase 1 clinical trial for pediatric patients (NCT02601937) and a phase 2 clinical trial for adults (NCT02601950) with relapsed rhabdoid tumors, synovial sarcomas, renal medullary carcinoma and epithelioid sarcoma [5]. In 2015,the selective and orally available EZH2 inhibitor, EPZ011989, was also reported and shown to have significant effects in the mouse xenograft model of B cell lymphoma [93]. Another more recent study introduced that ZLD1039, a highly selective, potent and orally available agent can decrease H3K27 methylation to upregulate tumor suppressor genes in breast cancer and suppress the tumor growth and metastasis in a mouse breast cancer xenograft model [94].

Recent studies demonstrated that inhibition of the interaction between EZH2 with PRC2 complex may also be a specific approach with efficacy against various neoplastic diseases with decreased toxicity. Kim et al. reported that stabilized alpha-helix of EZH2 (SAH-EZH2) peptides selectively inhibited H3K27me3 by disrupting the EZH2-EED complex and reducing EZH2 protein levels, thereby leading to growth arrest and differentiation of MLL-AF9 leukemic cells [95]. Similarly, the small molecule inhibitor Astemizole can also inhibit EZH2/EED interaction which destabilizes PRC2 complex thereby decreasing the proliferation of lymphoma cells [96]. Another inhibitor CPI-1205, disturbing the interaction of EZH2 with PRC2 complex, is currently evaluated in a phase 1 clinical trial in patients with B-cell lymphomas (NCT02395601) [97].

In addition to the competitive inhibitors of EZH2 expression and activity, a novel strategy to suppress EZH2 by protein degradation has been developed by Wang et al. They reported that a gambogenic acid (GNA) derivative, GNA022, specifically and covalently binds to Cys668 within the EZH2-SET domain, subsequently triggersEZH2 degradation through COOH terminus of Hsp70-interacting protein (CHIP)-mediated ubiquitination of EZH2, enhancingEZH2 degradation and inhibiting tumor growth [98].

## Conclusions

In conclusion, EZH2 is a methyltransferase and catalytic component of the PRC2, and mediates H3K27me3. Accumulating evidence from in vitro and in vivo models together with clinical studies collectively suggest that EZH2 is involved in the development and progression of several human malignancies. EZH2 activity is upregulated in cancer due to gain-of-function mutations, and overexpression by transcriptional, post-transcriptional and post-translational regulation. EZH2 downregulates expression of tumor suppressor genes and upregulates oncogenes, promoting cancer cell survival, proliferation, epithelial to mesenchymal and invasion. In particular, EZH2 has been also reported to be associated with drug resistance to chemotherapy and targeted therapy. Therefore, several targeted compounds inhibiting EZH2 expression and activity have been introduced in cancer therapeutics with promising results in pre-clinical studies combination with traditional chemotherapy with or not, and are currently under evaluation in phase 1 and 2 clinical trials.

However, the tumor suppressive roles of EZH2 were also identified that suppression of EZH2 promotes cancer progression in some cancer types, such as T-ALL, pancreatic cancer and clear cell renal carcinoma. Besides, recent studies also revealed that EZH2had complicated roles in tumor immunity. Inhibition of EZH2 in HSPCs gives rise to increased NK precursors and mature progeny displaying enhanced cytotoxicity against tumor cells. But EZH2 is essential for tumor-specific T cells-mediated tumor surveillance. EZH2 has also been shown to regulate the function of Tregs, dendritic cells and macrophage, all of which are critical parts of tumor microenvironment. Therefore, therapeutic targeting EZH2 is posing new challenges and worth future further investigation.

## Abbreviations

AML: acute myeloid leukemia
AR: androgen receptor
CcRCC: clear cell renal cell carcinoma
CDK1/2: Cyclin-dependent kinases 1 and 2
CHIP: COOH terminus of Hsp70-interacting protein
CPEB1: cytoplasmic polyadenylation element-binding protein 1
CRC: colorectal cancer
CRPC: castration-resistant prostate cancer
DAB2IP: Disabled Homolog2-Interacting Protein
DCs: dendritic cells
DHHC: Asp-His-His-Cys
DLBCL: diffuse large B-cell lymphoma
DNMT1: DNA methyltransferase 1
EZH2: Enhancer of zeste homolog 2
FoxM1: Forkhead box M1
GNA: gambogenic acid
GSCs: glioblastoma stem-like cells
H2AK-119ub1: histone H2A at lysine 119
H3K27me3: tri-methylation of histone H3 at Lys 27
HMT: histone methyl transferase
HNSCC: head and neck squamous cell carcinoma
HSPCs: hematopoietic stem and progenitor cells
MDS: myelodysplastic syndrome
MPN: myeloproliferative neoplasms
NEK2: NIMA-related kinase 2
NK: natural killer
PARP: poly (ADP-ribose) polymerase
PARPi: PARP inhibitors
PAT: protein acyltransferases
PcGs: Polycomb group proteins
PRC1: polycomb repressive complex 1
PRC2: polycomb repressive complex 2
pT416-EZH2: phosphorylates EZH2 at T416
RORα: RAR-related orphan receptor alpha
RTKi: receptor tyrosine kinase inhibitors
SAH-EZH2: stabilized alpha-helix of EZH2
T-ALL: T cell acute lymphoblastic leukemia
TAMs: tumor-associated macrophages
Th1: T helper 1
TKIs: tyrosine kinase inhibitors
TNBC: triple-negative breast cancer
Tregs: regulatory T cells
ZDHHC: zinc finger DHHC
